# Gender as a determinant of health in under-five children in Ethiopia; a secondary data analysis from EDHS 2016

**DOI:** 10.21203/rs.3.rs-2684226/v1

**Published:** 2023-05-31

**Authors:** Elbet Ketema, Saria Hassen

**Affiliations:** Addis Ababa University; Emory University

**Keywords:** Gender, Under-five mortality, Ethiopian demographic health survey 2016

## Abstract

**Background:**

Under-five mortality is one of the key sustainable development goal targets. Despite the great strides made globally, Under-five mortality remains high in many developing countries like Ethiopia. Child health status is determined by a myriad of factors at the individual, family and community level, furthermore, a child’s gender has been shown to affect the probability of infant and child mortality.

**Methods:**

A secondary data analysis was conducted using Ethiopian demographic health survey 2016 to assess association between gender and under-five child health. A representative sample of 18,008 households was selected. After data cleaning and entry, analysis was done using the Statistical Package for Social Sciences (SPSS) version 23. Uni-variable and multivariable logistic regression model were employed to determine the association between under-five child health and gender. In the final multivariable logistic regression model, the association of gender with childhood mortality was declared statistically significant at P value < 0.05.

**Result:**

A total of 2,075 under five children from EDHS 2016 were included in the analysis. Majority (92%) were rural dwellers. More male children were found to be underweight (53% Vs 47%) and wasted (56.2% Vs 43.8%) compared to female children. A higher proportions of females were vaccinated (52.2%) compared to 47.8% in males. Health seeking behavior for fever (54.4%) and diarrheal diseases (51.6%) were also found to be higher for females. However, in a multivariable logistic regression model, there was no statistically significant association found between gender and under-five child children health measures.

**Conclusion:**

Although it was not statistically significant association, females were found to have a better health and nutritional outcomes compared to boys in our study.

## Introduction

Improving child survival remains a matter of urgent concern. In 2019 alone, roughly 14,000 under-five deaths occurred worldwide every day which is an intolerably high number of largely preventable child deaths (1).

This burden of child mortality is unevenly distributed throughout the world. Over 70% of all under-five death occurs in Africa and south East Asia. One in twelve children in sub Saharan Africa dies before their fifth birthday which is 15 times higher than the risk for children born in high-income countries and 20 years behind the world average (1).

Ethiopia is one of the hardest hit countries with an alarming number of child deaths. In the country 1 in 15 children die before reaching age 5, and 7 in 10 of the deaths occur during infancy (2).

To achieve the sustainable development goal (SDG) target of under-five mortality rate of 25 or fewer deaths per 1000 live births by 2030, a total of 47 countries need to increase their pace of progress including Ethiopia (3).

Child health status is determined by a myriad of factors at the individual, family and community levels. A child’s gender has been shown to affect the probability of infant and child mortality. Owing to biological factors, male infants have a higher risk of mortality during the first year of life. In addition differential treatment of boys and girls, owing to cultural and socioeconomic factors also affect the chances of survival during childhood (4).

Gender is a major determinant of health and as such should be properly understood and thoroughly investigated. Although gender inequality in child health has been consistently reported throughout the world, there is still substantial knowledge gap on how gender mediates child health in Ethiopia. In addition implementation of interventions to mitigate gender inequalities that hinder child health requires additional perspective and research.

This study seeks to evaluate gender as a determinant of under-five health in Ethiopia and explore factors associated with it. This work will inform the disease prevention and control strategy and serve as a base line assessment laying the foundation to devise policies and programs to address gender inequality in health during childhood in Ethiopia.

## Method and Materials

Administratively, regions in Ethiopia are divided into zones, and subsequently into administrative units called weredas. Each wereda is further subdivided into the lowest administrative unit, called kebele. During the 2007 census each kebele was subdivided into census enumeration areas (EAs), which were convenient for the implementation of the census. The data source for this analysis is the 2016 Ethiopian DHS which was undertaken over a 5-months period from 18 January, 2016 to 27 June, 2016 which was designed to provide population and health indicators at the national (urban and rural) and regional levels. The data is publicly available at this site (http://www.measuredhs.com/data/available-datasets.cfm). The 2007 Population and Housing Census, conducted by the central statistical agency (CSA), provided the sampling frame from which the 2016 EDHS sample was drawn. The sample was selected using a stratified, two stage cluster design and EAs were the sampling units for the first stage. In the second stage, a fixed number of 28 household per cluster were selected with an equal probability systematic selection from the newly created household listing. The sample included 645 EAs, 202 in urban areas and 443 in rural areas. Households comprised the second stage of sampling. A complete listing of households was carried out in each of the 645 selected EAs from September to December 2015. A representative sample of 18,008 households was selected (EDHS 2016).

### Dependent variable (Outcome of interest):

Under-five mortality, vaccinations, seeking care in past 2 weeks for ARI/fever/diarrhea, stunted growth, under weight, exclusive breastfeeding, and anemia.

### Independent variable:

Gender of the child.

Gender of the child is defined as the socially constructed characteristics of a boy and a girl.

### Covariates

Based on literature review, the following covariates were selected:

Parental wealth index, birth order, and number of children in household, age of the mother, place of residence, region, Parental educational level, mode of delivery, antenatal care check-up, place of delivery, toilet facility, source of drinking water.

#### Data analysis and Interpretation

After data cleaning and entry, analysis was done using the Statistical Package for Social Sciences (SPSS) version 23. Descriptive statistics and cross tabulation was performed to describe the study variables. Bivariate association between each child’s gender was first examined using chi square test. Univariable and multivariable logistic regression models were employed to determine the association between under-five child health and gender. Variance inflation factor was used to assess presence of co linearity. Crudes odds ratio (COR) and adjusted odds ratio (AOR) was presented with 95% confidence interval. Each covariate was included in the multivariable model regardless of their statistical significance in the uni-variable analysis. In the final multivariable logistic regression model, the association of gender with childhood mortality was declared statistically significant if p-value < 0.05. Sample weights that account for complex survey design and unequal probabilities of selection were incorporated in all the analysis.

## Results

### 1. Socio-demographic characteristics

A total of 2,075 under five children from EDHS 2016 were included in the analysis. The majority (92%) were rural dwellers. More males (57%) were from the urban area when compared to females (43%) but parents of males and females had a similar wealth index. In both males and females there were more infants than any other age group. Boys had relatively younger (54.7% Vs 45.3%) and uneducated mothers (51% Vs 49%) compared with girls (Table 1).

### 2. Under five child health

In this survey, 38% of under-five children were stunted, 24% underweight and 10% wasted. However, more male children were underweight (53% Vs 47%) and wasted (56.2% Vs 43.8%) compared to female children. The proportion of male and female children who were stunted was similar (50.8% Vs 49.2%). Around 52.2% of under-five children who were at least vaccinated once were females compared to a 47.8% of males. Female children were also found to have higher health seeking for fever (54.4%) and diarrheal diseases (51.6%) than males (45.6% and 48.4$) respectively. More than half (57%) of children aged 6–59 months in this survey were anemic. There were more females with severe anemia (55.3%) when compared to males (44.7%) (Table 2).

### 3. Gender and under five child health

Among 2,075 under five children evaluated in this study, there was no statistically significant association found between gender of a child and under-five children health outcomes. The odds of being wasted was three times more in females compared to males, AOR = 3.09 (CI 0.50, 18.89). The odds of care seeking behavior for fever was also 2.53 AOR = (CI 0.61, 10.48) times more in females when compared to boys. Similarly the odds of being exclusively breast fed was AOR = 2.79(CI 0.70, 11.02) times more in females. Again, the odds of being vaccinated among female under fives was AOR = 1.27(CI 0.47, 3.39) times those of males. However, these associations were not found to be statistically significant. Females were also found to be less stunted and underweight when compared to males with odds ratio of AOR = 0.48(0.16, 1.41) and AOR = 0.36 (0.086, 1.54) respectively (Table 3).

## Discussion

Child health remains a global health priority (3). In Ethiopia, under-five mortality is declining steadily but still remains high (5). There are a myriad of factors determining child health including gender (6). In this study the EDHS 2016 was used to assess the association of under five child health parameters with gender. Our study did not find a statistically significant association between under-five child health and gender. The result of this study is unexpected since the majority of Ethiopians live in rural area where1905 samples out of 2070 in this study were from. The rural community is thought to be a very traditional community and females are usually disadvantaged.

Though there was no statistically significant association found in this study, females were found to be less stunted AOR of 0.48 (CI: 0.16, 1.41) and underweight AOR of 0.36(0.086, 1.54) compared to boys. This is contrary to one study in Bangladesh which showed a substantially higher prevalence of malnutrition among female children than male. In depth dietary surveys in the same study also found males to consistently consume more calories and proteins than females at all ages (9). A similar study from rural eastern Kenya were boys were found to have consistently higher energy intake than girls and more girls were found to be stunted, underweight and wasted (10). Contrary to the above studies, however, other studies have not found significant difference between girls and boys in terms of nutrition and health outcomes (12).

Exclusive breast feeding was also one of the parameters where girls were found to benefit more than boys in our study, although, this was not statistically significant. The odds of being exclusively breast fed was 1.35 times more common for females with COR = 1.35(CI 0.86, 2.13) and AOR = 2.79(CI 0.70, 11.02). Similarly, for sub-Saharan countries, the male breast feeding advantage is much smaller. In contrary, Boys are breast fed for 0.657 months longer than girls in North African countries (11).

In our study, statistically significant difference in care seeking for sick boys and girls during febrile and diarrheal diseases was not observed. However the odds of care seeking for sick boys and girls during diarrheal and febrile illness was AOR = 0.95(CI 0.26, 3.49) and AOR = 2.53(CI 0.61, 10.48) respectively. Our finding is similar to one meta-analysis which evaluated 57 countries and significant difference in care seeking for sick boys and girls were not observed in most countries (13).

In contrary to this study, females were found to die more than boys at the age of 5, 10, 15 and 20 in Egypt and the major cause of excess female mortality was attributed to the favored treatment boys received for digestive and respiratory illness (8). Similarly, utilization of health care services showed marked male preferences in Bangladesh (9).

A statistically significant difference in vaccination status was also not found between boys and girls in this study. The odds of being vaccinated among female was AOR = 1.27(CI 0.47, 3.39) when compared to boys. Similar to our study, in one systematic review, the pooled odds ratio for sex did not show significant differences between girls and boys in vaccination out-come (14). This is in contrary to one study in India which was conducted among 4000 children between the age of 1 and 2, the likeli-hood of female being fully vaccinated was 5% less than that for boys. In the same study, in certain subgroups of children, especially children from poorest households, boys were more likely to not being vaccinated than girls (7).

## Conclusion

Ethiopia is considered a culturally conservative country rooted in traditional beliefs among them priority given to sons over daughters. Yet, there was no statistically significant association between gender of a child and under five child health based on our analysis. Females were found to have a better health and nutritional outcomes compared to boys in our study. This can be in part explained by the higher number of boys with young and uneducated mothers.

## Figures and Tables

**Table 1:** Frequency table of characteristics of the study sample by gender in Ethiopia, EDHS 2016 (n = 2075)

Variables	Gender		p-value
	
	Male	Female	
	N (%)	N (%)	

**Maternal age**	**164(54.7%)**	**148(45.3%)**	**0.441**
**<20**	**717(49.5%)**	**683(50.5%)**	
**20–34**	**134(45.6%)**	**147(54.4%)**	
**35+**			

**Wealth index**	507(49.7%)	473(50.3%)	0.991
Poorest	196(50.6%)	196(49.4%)	
Poorer	149(49.3%)	147(49.3%)	
Middle	114(50.5%)	113(49.5%)	
Richer	93(47.9%)	87(52.1%)	
Richest			

**Place of residence**	87(57.0%)	78(43.0%)	0.241
Urban	972(49.5%)	938(50.5%)	
Rural			

**Education level of mother**	761(50.4%)	707(49.6%)	0.914
No education	238(48.5%)	253(51.5%)	
Primary	45(47.7%)	44(52.3%)	
Secondary	15(56.6%)	12(43.4%)	
Higher			

**Age of the child in month**	306(49.0%)	303(51.0%)	0.940
Under 6 months	256(50.5%)	224(49.5%)	
6–11 months	497(50.0%)	489(50.0%)	
12–23 months			

**Number of under 5 in house hold**	312(50.5%)	319(49.5%)	0.780
1	504(51.8%)	462(48.2%)	
2	210(44.2%)	196(55.8%)	
3	27(52.2%)	31 (47.8%)	
4	3(3.7%)	5(96.3%)	
5	3(50.1%)	3(49.9%)	
6			

**Table 2:** Characteristics of mother-infant pairs in Ethiopia, EDHS 2016 (n = 2075)

Variable	Gender		p-value
	
	Male	Female	
	N (%)	N (%)	

**Stunting**	**245(50.8%)**	**211(49.2%)**	**0.571**
**Yes**	**711(48.7%)**	**724(51.3%)**	
**No**			

**Underweight**	265(53.0%)	231(47.0%)	0.221
Yes	746(48.8%)	755(51.2%)	
No			

**Wasting**	171(56.2%)	138(43.8%)	0.101
Yes	843(48.9%)	851(51.1%)	
No			

**Toilet facility**	587(53.3%)	526(46.7%)	0.139
Open defecation	365(47.5%)	398(52.5%)	
Un improved facility	107(46.2%)	92(53.8%)	
Improved facility			

**Water source**	557(51.5%)	507(48.5%)	0.276
Improved	502(48.2%)	509(51.8%)	
Unimproved			

**ANC visit**	488(50.8%)	440(49.2%)	0.735
No ANC visits	319(50.0%)	304(50.0%)	
1 to 3 visits	252(47.8%)	272(52.2%)	
More than 3 visits			

**Place of delivery**	771 (48.8%)	773(51.2%)	0.264
Home	288(53.0%)	243(47.0%)	
Health facility			

**Delivery by caesarean section**	18(60.1%)	12(39.9%)	0.465
Yes	1041(49.7%)	1004(50.3%)	
No			

**Ever vacdnated**	624(47.8%)	597(52.2%)	0.186
Yes	435(52.7%)	419(47.3%)	
No			

**Diarrhea medical treatment**	71(48.4%)	66(51.6%)	0.398
Yes	82(55.5%)	66(44.5%)	
No			

**Fever/cough medical treatment**	65(45.6%)	59(54.4%)	0.995
Yes	165(53.1%)	145(46.9%)	
No			

**Stunting**	**245(50.8%)**	**211(49.2%)**	**0.571**
**Yes**	**711(48.7%)**	**724(51.3%)**	
**No**			

**Anemia**	23(44.7%)	28(55.3%)	0.356
Severe	115(55.0%)	106(54.0%)	
Moderate	275(45.1%)	265(54.9%)	
Mild	646(51.1%)	617(48.9%)	
Not anemic			

**Currently breast feeding**	920(50.3%)	872(49.7%)	0.408
Yes	139(46.3%)	144(53.7%)	
No			

**Given child anything other than breast milk**	214(56.8%)	156(43.2%)	0.187
Yes	816(49.2%)	837(50.8%)	
No			

**Table 3:** Association between Gender and vaccination, care seeking in the past 2 weeks, stunting, underweight, wasting, anemia and exclusive breast feeding among children aged less than five years in Ethiopia, EDHS 2016.

	Stunting		Wasting		Underweight	
**Gender**	**Yes**	**No COR(95% CI) AOR(95% CI)**	**Yes**	**No COR (95% CI) AOR(95% CI)**	**Yes**	**No COR (95% CI) AOR(95% CI)**
Male	245	711 1.00 1.00	171	843 1.00 1.00	265	746 1.00 1.00
Female	211	724 0.92(0.68,1.22) 0.48(0.16, 1.41)	138	851 0.74(0.52,1.06) 3.09(0.50, 18.89)	231	755 0.84(0.64, 1.1) 0.36(0.08, 1.54)


	Anemia		Health seeking for diarrhea		Health seeking for fever	
**Gender**	**Yes**	**No COR(95% CI) AOR(95% CI)**	**Yes**	**No COR (95% CI) AOR(95% CI)**	**Yes**	**No COR (95% CI) AOR(95% CI)**
Male	413	646 1.00 1.00	71	82 1.00 1.00	65	165 1.00 1.00
Female	399	617 1.16(0.92, 1.47) 0.65(0.19,2.1)	66	66 1.32(0.67,2.59) 0.95(0.26, 3.49)	59	145 1.35(0.76, 2.39) 2.53(0.61, 10)

	Exclusive breast feeding		Vaccination status	
**Gender**	**Yes**	**No COR(95% CI) AOR(95% CI)**	**Yes**	**No COR (95% CI) AOR(95% CI)**
Male	816	214 1.00 1.00	624	435 1.00 1.00
Female	837	156 1.35(0.86, 2.13) 2.79(0.70, 11)	595	419 1.21(0.91, 1.62) 1.27(0.47, 3.3)

## Data Availability

The datasets used and/or analyzed during the current study are available from the corresponding author on reasonable request. https://dhsprogram.com/data/dataset/Ethiopia_Standard-DHS_2016.cfm?flag=0

## Abbreviations

EDHS: Ethiopian demographic health survey
SDG: Sustainable development goals
WHO: World health organization
UNICEF: United Nations children’s emergency fund
